# Etanercept in the treatment of ankylosing spondylitis: A systematic review and meta-analysis

**DOI:** 10.3892/etm.2014.1974

**Published:** 2014-09-17

**Authors:** YA-FEI LIU, HUI DONG, SHENG-HAO TU, CUI-HONG ZHENG, PEI-LIN LIU, YONG-HONG HU

**Affiliations:** Department of Integrated Traditional Chinese and Western Medicine, Tongji Hospital, Tongji Medical College, Huazhong University of Science and Technology, Wuhan, Hubei 430030, P.R. China

**Keywords:** etanercept, ankylosing spondylitis, systematic review, meta-analysis

## Abstract

Etanercept (ETN) has been widely applied in the treatment of ankylosing spondylitis (AS). As the use of ETN has increased, associated adverse effects have been reported frequently. Previous meta-analyses have focused on comparing the differences in clinical outcomes between ETN and placebo (PBO). The present meta-analysis evaluated randomised controlled trials (RCTs) to compare the effects of ETN and a PBO or sulfasalazine (SSZ) in patients with AS. The study population characteristics and the main results, including the Assessment in AS 20% response (ASAS 20), the Bath AS Disease Activity Index (BASDAI) and the Bath AS Functional Index (BASFI), were extracted. The pooled odds ratios (ORs) or weighted mean differences (MDs) were calculated using a fixed or random effects model. Fifteen randomised controlled trials (RCTs) involving 2,194 subjects were included. Compared with a PBO, ETN significantly improved the ASAS 20 [P<0.00001; OR, 8.25; 95% confidence interval (CI), 5.92–11.50], BASDAI (P<0.00001; MD, −18.81; 95% CI, −24.47 to −13.15) and BASFI (P<0.00001; standard MD, −0.68; 95% CI, −0.85 to −0.50). In comparison with SSZ, ETN significantly decreased the BASDAI (P<0.00001; MD, −2.40; 95% CI, −2.89 to −1.90) and C-reactive protein (CRP) levels (P<0.0001; MD, −8.01; 95% CI, −11.73 to −4.29). The most common adverse effect of ETN was an injection site reaction. This meta-analysis shows that ETN monotherapy is effective in improving physical function and reducing disease activity in patients with AS. Compared with SSZ, ETN markedly decreased the BASDAI and CRP levels. However, the efficacy of ETN in treating AS requires further evaluation by more RCTs in a larger population of patients prior to recommending ETN as a substitute for synthetic disease-modifying antirheumatic drug (DMARD) monotherapy, or combinations of synthetic DMARDs.

## Introduction

Ankylosing spondylitis (AS) is characterised by inflammatory back pain, asymmetrical peripheral arthritis, enthesitis and extra-articular features (1). AS is genetically associated with human leucocyte antigen B27, and the average age at disease onset is 27.7 years in B27^−^ AS and 24.8 years in B27^+^ AS (2). The worldwide prevalence of AS varies from 0.1 to 1.1% in the adult Caucasian population (3). Patients with AS are likely to lose their physical function and ability to work, which is likely to have a significant impact on the quality of life unless an appropriate treatment is administered to the patients.

Tumour necrosis factor-α (TNF-α) plays a crucial role in the pathogenesis of AS, and TNF-α concentrations are increased in the circulation (4) and synovial tissue (5) in patients with AS. Etanercept (ETN), which binds to TNF-α and blocks its biological activity, is a recombinant, dimeric fusion protein. It has been demonstrated that ETN is beneficial in the treatment of rheumatic diseases, including rheumatoid arthritis (6), psoriatic arthritis (7) and polyarticular juvenile rheumatoid arthritis (8). However, a number of patients with AS are not suitable for ETN treatment due to side-effects or a poor clinical response. Owing to the high medication cost, ETN treatment is not generally favoured in the developing world.

Non-steroidal anti-inflammatory drugs (NSAIDs) are the first-line drugs for AS. Conventional disease-modifying antirheumatic drugs (DMARDs) are occasionally recommended by physicians; however, there is no evidence for the efficacy of these drugs in the treatment of axial disease (9). At an early disease stage, patients with AS with a higher erythrocyte sedimentation rate (ESR) and peripheral arthritis may benefit from sulfasalazine (SSZ), as concluded in a Cochrane review (10). Another meta-analysis (11) also indicated that SSZ was a safe and effective drug for the short-term treatment of AS. SSZ may be considered in patients with peripheral arthritis, based on the Assessment in AS (ASAS)/European League Against Rheumatism recommendations (9). In addition, SSZ has the advantage over ETN in medical cost. Therefore, SSZ has been extensively used in underdeveloped countries.

To the best of our knowledge, Li *et al* (12) performed the first systematic review concerning the effects of ETN in the treatment of AS in 2009. Subsequent systematic reviews have been reported. However, the previous reviews concentrated on comparing the total effects of ETN. In the present study, two subgroup analyses were performed to compare the effects of ETN with a placebo (PBO) or SSZ. Continuous updates can collect data from new studies. Thus, a novel comprehensive systematic review and overall meta-analysis are requisite for drawing more reliable conclusions about the effects of ETN in the treatment of AS.

## Methods

To ensure the accuracy of the present systematic review and meta-analysis, the results were designed and reported by employing a checklist of items that was as consistent as possible with the Preferred Reporting Items for Systematic Review and Meta-Analyses statement (13).

### Literature search strategy

The following digital databases were searched for the identification of studies: PubMed, Embase, the Cochrane Library and ClinicalTrials.gov. In addition, Chinese databases were searched, including the China National Knowledge Infrastructure, VIP, Chinese Biomedical Literature and WanFang Databases, and the Chinese Clinical Trial Register. All the databases were searched from the available date of inception to the latest issue (2013).

Different search strategies were merged as follows. For the English databases, free-text terms were used, including ‘Etanercept’, or ‘Enbrel’ and ‘Ankylosing Spondylitis’, ‘AS’ or ‘Bechterew Disease’. For the Chinese databases, free-text terms were used, such as ‘Yi Na Xi Pu’, ‘Yi SaiPu’, ‘En Li’ or ‘QiangKe’ (which are the alternative names of ETN in Chinese) and ‘QiangZhi Xing Ji Zhu Yan’ or ‘QiangZhi Xing JiZhui Yan’ (which means ‘AS’ in Chinese). A filter for clinical trials was applied. To collect an adequate number of trials, the reference lists of the relevant publications were carefully read.

### Inclusion and exclusion criteria

Regardless of blinding, publication status or language, RCTs were included. Individual cases that were diagnosed as exhibiting AS according to the modified New York criteria for AS (14) were collected. For the types of interventions, treatment with ETN alone in RCTs was considered. The control groups consisted of treatments with a PBO or SSZ. Studies were only included if the intervention was administered for at least six weeks.

Case reports, reviews, retrospective studies, open-label extension studies and studies without a control group were excluded. Also excluded were RCTs without a clear description of the required outcomes of interest, and particularly those studies that did not describe the exact means and standard deviations (SDs) of the outcomes. The studies comparing various TNF inhibitors were also excluded since there is no evidence to support a difference in their efficacies in treating the axial and articular/entheseal disease manifestations (9).

### Data extraction

The search strategy, application of inclusion criteria, data extraction and statistical analyses were independently executed by two of the present authors. Any disagreements were resolved by consensus or mediation by a third author. The methodological quality of each study was assessed according to the following criteria: Baseline difference, method of randomisation, degree of blinding, use of intention-to-treat (ITT) analysis, and description of dropouts and withdrawals. The validated Jadad scale was used to assess the quality of each study (15).

The primary outcome was the proportion of patients achieving the ASAS 20% response (ASAS 20) established by the ASAS Working Group (16). The secondary outcomes comprised the ASAS 50, ASAS 70, Bath AS Disease Activity Index (BASDAI) (17), BASDAI 50, Bath AS Functional Index (BASFI) (18), ASAS partial remission (ASAS PR) (16), and levels of ESR and C-reactive protein. Spinal mobility, assessed by the Schober’s test (ST) and the occiput-to-wall (OW) distance, was also considered to be a secondary outcome. The authors of the study were contacted if any outcome was ambiguous or absent from the article. If the author could not be reached, the data was extracted by consensus.

### Statistical analysis

To summarise the effects of ETN, Review Manager statistical software (version 5.2; Cochrane Collaboration, Copenhagen, Denmark) was used to calculate weighted mean differences (MDs), standard MDs (SMDs), and the 95% confidence interval (CI) for the continuous data. MDs were used if outcomes were measured in the same way between trials, while SMDs were used if the same outcomes were measured by adopting different methods. For dichotomous data, the data were pooled and expressed as odds ratios (ORs) with a 95% CI. Heterogeneity was evaluated via subgroup analysis using the χ^2^ and I^2^ tests. Where the heterogeneity test was at P>0.10, the data were pooled via a fixed effects model; otherwise, a random effects model was used. Publication bias was evaluated by the Egger’s regression asymmetry and Begg’s tests when the number of included trials exceeded five (Stata 12.0 software; StataCorp LP, TX, USA). The total effect was tested using a Z score, and P<0.05 was considered to be statistically significant in all the analyses. To minimise the clinical heterogeneity, two subgroup analyses were performed: ETN compared with a PBO and ETN compared with SSZ.

## Results

### Study characteristics

**Figure 1:**
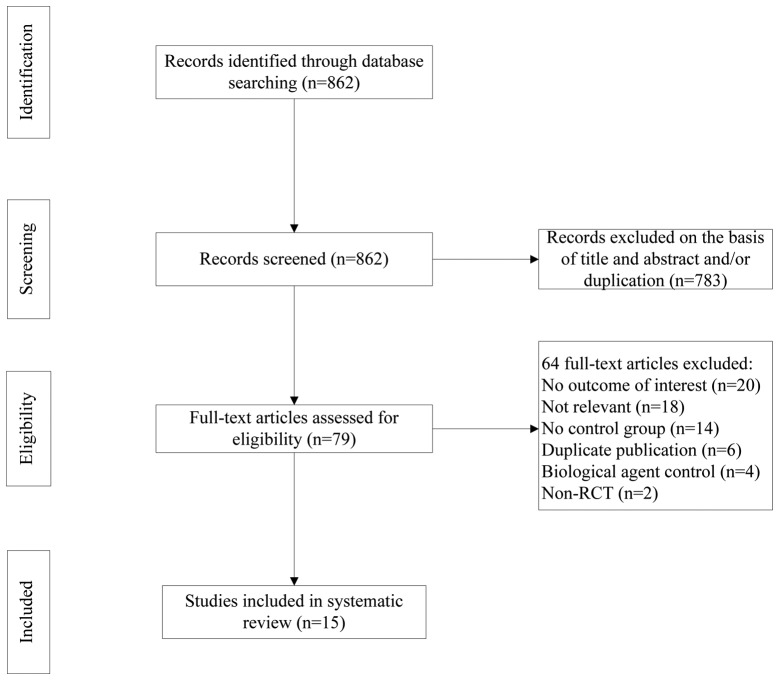
Study selection flow chart. RCT, randomised controlled trial.

The process of study selection is shown in Fig. 1. As shown in Table I, the included studies were published as full text between 2002 and 2011. Seven studies were published in Chinese, and eight studies (19–26) were published in English. Nine studies (19,20,22–28) were multicentre trials, whereas the remaining six were performed at a single centre. Together, those trials included a total of 2,194 participants.

In addition to an initial double-blind trial, five included trials (20,27–30) conducted a subsequent open-label extension study in which the two groups were treated with ETN (50 mg) per week. In the trial reported by Zhao *et al* (31), the dose of ETN was reduced to 25 mg per week after seven weeks. The trial conducted by Brandt *et al* (26) had two phases: an initial PBO-controlled period and an observational phase; only the PBO-controlled period was selected for analysis. The majority of the data in the study by Gorman *et al* (21) were expressed as the median ± SD and were discarded.

The dose of ETN applied in the included trials was 25 mg twice weekly (BIW) or 50 mg once weekly (QW). In the study reported by van der Heijde *et al* (25), three parallel groups were established, which were ETN (50 mg QW), ETN (25 mg BIW) and PBO; however, only ETN (25 mg BIW) was compared with the PBO. SSZ intake ranged from 1.0 to 3.0 g per day. The duration of interventions in the included trials was also different, ranging from 6 to 24 weeks. To minimise heterogeneity, when compared with a PBO, the reported outcomes of the ASAS 20, ASAS 50 and ASAS 70 were measured at six weeks, with the exception of one trial (24) from which the data was extracted at eight weeks. Twelve trials performed an ITT analysis, two (27,32) performed a treated-per-protocol (TPP) analysis, and one (28) performed an ITT and TPP analysis.

### Quality of the included studies

Approximately three-quarters of included trials were moderate quality (Jadad score ≥3), whereas four of the Chinese trials were of low quality (Jadad score <3) due to unclear randomisation, deficient allocation concealment, inadequate blinding, and undisclosed withdrawal and dropouts. Two subgroup analyses were performed to minimise the clinical heterogeneity.

### Publication bias

**Figure 2:**
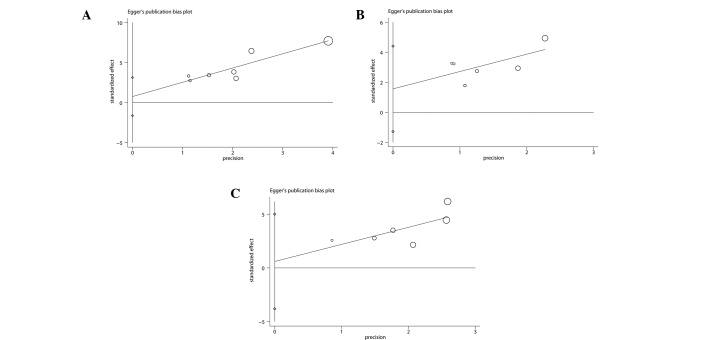
Publication bias in the included trials. Egger’s linear regression test for detecting publication bias in terms of (A) ASAS 20, (B) ASAS 50 and (C) Bath ankylosing spondylitis disease activity index 50. ‘○’ is a size graph symbol for the weight of each included study. The distance between two diamonds on the second vertical bar on the left represents the 95% confidence interval for the intercept. ASAS, assessment in ankylosing spondylitis.

The Egger’s publication bias plots and Begg’s test showed that there were no significant publication biases for three outcomes with number of included trials ≥6 (Fig. 2). However, the results cannot be regarded as convincing since three outcomes have <10 trials; the power of a funnel plot is considered to be restricted unless substantial bias is present and the number of trials is ≥10 (33).

### ETN compared with a PBO

**Figure 3:**
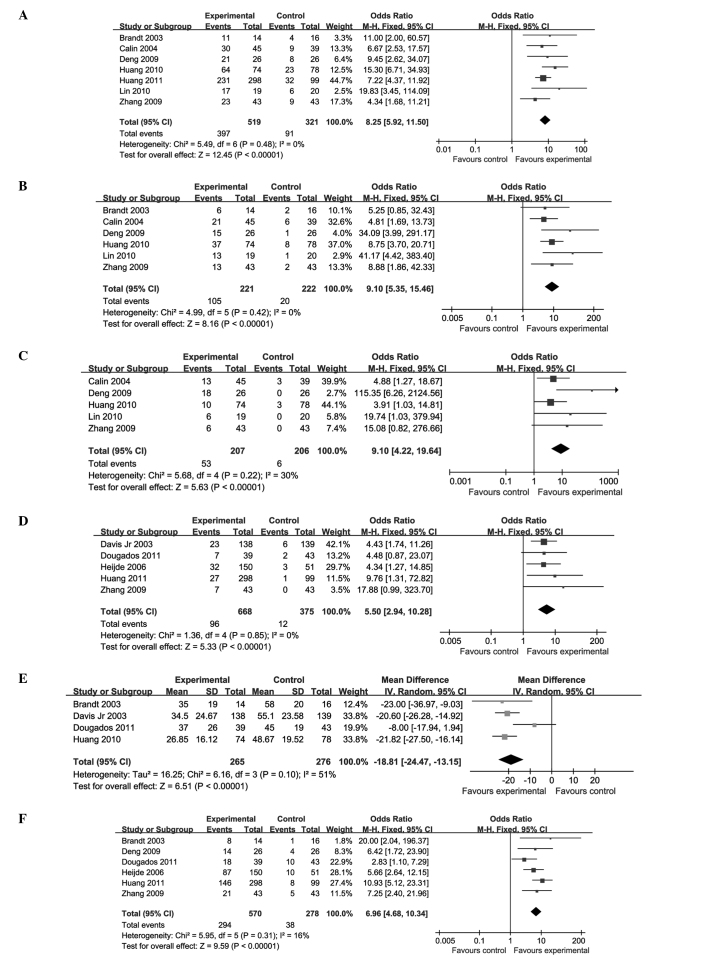

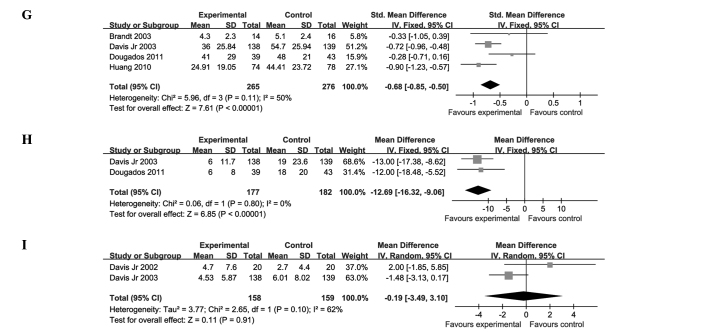
Forest plots of etanercept treatment compared with a placebo in terms of: (A) ASAS 20, (B) ASAS 50, (C) ASAS 70, (D) ASAS partial remission (E) BASDAI and (F) BASDAI 50. BASDAI, Bath ankylosing spondylitis disease activity index; ASAS, assessments in ankylosing spondylitis; CI, confidence interval; SD standard deviation. Forest plots of etanercept treatment compared with a placebo in terms of: (G) Bath ankylosing spondylitis functional index, (H) C-reactive protein and (I) occiput-to-wall. CI, confidence interval; SD standard deviation.

Eleven trials (involving 1,443 patients) compared the therapeutic effects of ETN and a PBO (19–22,24–30). The number of trial participants ranged from 14 to 300, and the trial duration varied from 6 to 24 weeks. There was no statistical heterogeneity between the studies. The pooled results displayed a significant difference between the ETN-treated and PBO groups, with the exception of the OW distance (P=0.91; MD, −0.19; 95% CI, −3.49–3.10). The ETN group was superior to the PBO group in terms of improvements in the ASAS 20 (P<0.00001; OR, 8.25; 95% CI, 5.92–11.50), ASAS 50 (P<0.00001; OR, 9.10; 95% CI, 5.35–15.46), ASAS 70 (P<0.00001; OR, 9.10; 95% CI, 4.22–19.64), BASDAI (P<0.00001; MD, −18.81; 95% CI, −24.47 to −13.15) and ASAS PR (P<0.00001; OR, 5.50; 95% CI, 2.94–10.28). In addition, the BASDAI 50 (P<0.00001; OR, 6.96; 95% CI, 4.68–10.34) was significantly enhanced and the BASFI (P<0.00001; SMD, −0.68; 95% CI, −0.85 to −0.50) and CRP (P<0.00001; MD, −12.69; 95% CI, −16.32 to −9.06) levels were decreased (Fig. 3; Table II).

### ETN compared with SSZ

Four trials (involving 751 patients) compared ETN with SSZ (23,31,32,35). The number of trial participants ranged from 20 to 378, and the trial duration ranged from 6 to 16 weeks. In the combined results, there was evident statistical heterogeneity between the comparisons for the ASAS 20 (P<0.0001) and BASFI (P=0.0009), and particularly for the ESR (P<0.00001). The above outcomes are described separately in the present analysis, rather than being subjected to combined analyses.

In two of these trials (32,35), the difference in the ASAS 20 values between the ETN and SSZ groups was not reported. The other two trials (23,31) showed that ETN induced greater increases in the ASAS 20 levels than SSZ did. In two trials (31,35), the difference in the BASFI levels between the ETN and SSZ groups was not reported. The other two trials (23,32) indicated that ETN had a greater effect than SSZ in decreasing the BASFI levels. Two of the trials (23,35) did not compare the ESR levels in the ETN group with the levels in the SSZ group. The other two trials (31,32) revealed that ETN was more effective than SSZ in reducing the ESR levels.

**Figure 4:**
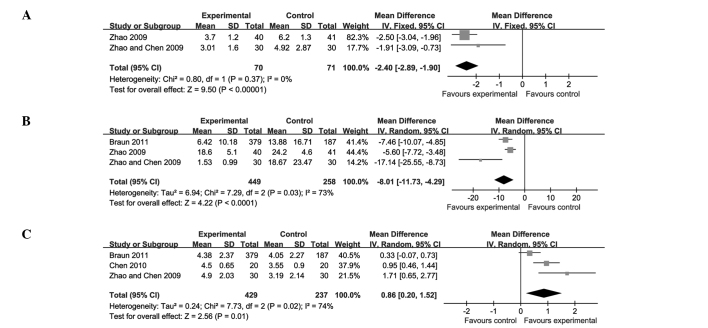
Forest plots of etanercept treatment compared with sulfasalazine in terms of (A) Bath ankylosing spondylitis disease activity index, (B) C-reactive protein and (C) Schober’s test. CI, confidence interval; SD, standard deviation.

There was a significant difference between ETN and SSZ in terms of the BASDAI (P<0.00001; MD, −2.40; 95% CI, −2.89 to −1.90) and CRP levels (P<0.0001; MD, −8.01; 95% CI, −11.73 to −4.29). A small but significant increase in the ST score (P=0.01; MD, 0.86; 95% CI, 0.20–1.52) was also found (Fig. 4; Table III).

### Adverse effects

Twelve of the 15 trials reported outcomes for adverse effects (AEs). The majority of the studies reported the incidence of an injection site reaction (ISR); however, one trial did not report the group in which the ISR occurred (29). Four trials reported abnormality of liver function in the ETN group (24,27,28,31), and in one trial, this was associated with concomitant indomethacin treatment (24). In the trials of Dougados *et al* (19) and Deng *et al* (29), neutropenia was detected during the ETN treatment. Non-neutralising anti-ETN antibodies were found in the ETN group in the trials of Davis Jr *et al* (22) and van der Heijde *et al* (25). In one study, one patient in the PBO group experienced aggravated AS, which resulted in the withdrawal of the patient from the study, and another patient in the ETN group withdrew due to a lung neoplasm (19). Two neurologic events were reported in a single patient treated with ETN: tinnitus and benign fasciculations (34). In the trial reported by Davis Jr *et al* (22), seven patients in the ETN group discontinued the study: five due to serious AEs and two due to gastrointestinal haemorrhage and ileitis. Additionally, one life-threatening event occurred (a suicide attempt in the PBO group) in the same trial. An ETN-treated patient with acute myocardial infarction underwent angioplasty but continued to participate in the study (24).

## Discussion

Although several systematic reviews reporting the efficacy and safety of ETN in the treatment of AS have been conducted, they focused on evaluating the differences between ETN and a PBO (12,36,37). Unlike the previous reviews, the present review study included 15 trials and set two subgroups to minimise heterogeneity. Furthermore, new studies were included that were published subsequent to the previous reviews. Thus, the present systematic review differs from the previous studies. For comparisons with a PBO group, the present results are consistent with those of previous reviews (12,36,37) in terms of increasing ASAS 20. In addition, these results are consistent with two previous reviews regarding the ASAS PR and BASDAI (12,37) between ETN-treated and PBO groups. Unlike one of the previous reviews (12), the present review shows that the ETN group exhibits reduced BASFI and CRP levels compared with the PBO group. Furthermore, the present study shows that compared with SSZ, ETN is capable of decreasing the BASDAI and CRP level and increasing the ST score. In addition, in the present review, it was identified that the AEs of ETN generally appeared to be mild or moderate. The most common side-effect was an ISR, and this reaction was tolerable for the majority of the participants.

Several limitations exist in this systematic review. Firstly, half of the included studies were conducted in Chinese populations, which implies a high risk of selection bias. Secondly, the majority of the studies published in Chinese were of poor quality. Three studies (31,32,35) did not use blinding and performed unclear allocation concealment. Therefore, potential bias, such as in the selection of patients, the administration of interventions and assessment of outcomes, could have resulted in the overestimation of the therapeutic efficacy of ETN. Finally, the limited number (two to seven) of trials included in each subgroup dampened the positive evidence for the efficacy of ETN in treating AS. Certain vital outcomes were not reported for the SSZ control. Consequently, it was not possible to draw a definitive conclusion concerning whether ETN monotherapy was improved compared with SSZ. Therefore, it is necessary for all outcomes to be carefully explained.

In conclusion, this meta-analysis suggests that ETN exhibits beneficial effects in terms of improving the ASAS 20, BASDAI and BASFI during the treatment of AS and exhibits superiority in comparison with the effects of a PBO. Due to the lack of high-quality clinical trials, whether ETN monotherapy is improved compared with SSZ in disease activity control and symptom relief remains to be validated. Therefore, it is necessary for large and well-designed RCTs to be performed prior to recommending the use of ETN to replace synthetic DMARD monotherapy or combinations of synthetic DMARDs.

## Figures and Tables

**Table I:** Characteristics of the included trials.

	Patients (n)		Intervention			
			
Study	Experimental	Control	Experimental	Control	Duration (weeks)	Outcomes
Brandt *et al* 2003 (26)	14	16	ETN	PBO	6	ASAS 20, ASAS 50, BASDAI, BASDAI 50, BASFI, AEs
Calin *et al* 2004 (24)	45	39	ETN	PBO	8	ASAS 20, ASAS 50, ASAS 70, AEs
Davis Jr *et al* 2003 (22)	138	139	ETN	PBO	24	ASAS PR, BASDAI, BASFI, OW, CRP, AEs
Gorman *et al* 2002 (21)	20	20	ETN	PBO	16	OW, AEs
Deng *et al* 2009 (29)	26	26	ETN	PBO	6	ASAS 20, ASAS 50, ASAS 70, BASDAI 50, AEs
Dougados *et al* 2011 (19)	39	43	ETN	PBO	12	BASDAI, BASDAI 50, BASFI, ASAS PR, CRP, AEs
van der Heijde *et al* 2006 (25)	150	51	ETN	PBO	12	BASDAI 50, ASAS PR, AEs
Huang *et al* 2010 (28)	74	78	ETN	PBO	6	ASAS 20, ASAS 50, ASAS 70, BASDAI, BASFI, AEs
Huang *et al* 2011 (27)	300	100	ETN	PBO	6	ASAS 20, BASDAI 50, ASAS PR, AEs
Lin *et al* 2010 (20)	19	20	ETN	PBO	6	ASAS 20, ASAS 50, ASAS 70
Zhang *et al* 2009 (30)	43	43	ETN	PBO	6	ASAS 20, ASAS 50, ASAS 70, BASDAI 50, ASAS PR
Braun *et al* 2011 (23)	378	187	ETN	SSZ	16	ASAS 20, BASFI, ST, CRP, AEs
Chen *et al* 2010 (35)	20	20	ETN	SSZ	12	ST
Zhao *et al* 2009 (31)	30	30	ETN	SSZ	6	ASAS 20, BASDAI, ST, ESR, CRP, AEs
Zhao *et al* 2009 (32)	43	43	ETN	SSZ	12	BASDAI, BASFI, ESR, CRP, AEs

ETN, etanercept; PBO, placebo; SSZ, sulfasalazine; ASAS, assessment in ankylosing spondylitis; BASDAI, Bath ankylosing spondylitis disease activity index; BASFI, Bath ankylosing spondylitis functional index; AE, adverse effect; OW, occiput-to-wall; ST, Schober’s test; PR, partial remission; ESR, erythrocyte sedimentation rate; CRP, C-reactive protein.

**Table II:** Results of the meta-analysis for the placebo control.

	Heterogeneity			Test for overall effect		
		
Outcomes	χ^2^	P-value	*I*^2^ (%)	Z	P-value	OR (95% CI)
ASAS 20	5.49	0.48	0	12.45	<0.00001	8.25 (5.92, 11.50)
ASAS 50	4.99	0.42	0	8.16	<0.00001	9.10 (5.35, 15.46)
ASAS 70	5.68	0.22	30	5.63	<0.00001	9.10 (4.22, 19.64)
BASDAI	6.16	0.10	51	6.51	<0.00001	−18.81 (−24.47, −13.15)
BASDAI 50	5.95	0.31	16	9.59	<0.00001	6.96 (4.68, 10.34)
BASFI	5.96	0.11	50	7.61	<0.00001	−0.68 (−0.85, −0.50)
ASAS PR	1.36	0.85	0	5.33	<0.00001	5.50 (2.94, 10.28)
CRP	0.06	0.80	0	6.85	<0.00001	−12.69 (−16.32, −9.06)
OW	2.65	0.10	62	0.11	0.91	−0.19 (−3.49, 3.10)

ASAS, assessment in ankylosing spondylitis; BASDAI, Bath ankylosing spondylitis disease activity index; BASFI, Bath ankylosing spondylitis functional index; OW, occiput-to-wall; PR, partial remission; CRP, C-reactive protein; OR, odds ratio; CI, confidence interval.

**Table III:** Results of meta-analysis for the sulfasalazine control.

	Heterogeneity			Test for overall effect		
		
Outcomes	χ^2^	P-value	*I*^2^ (%)	Z	P-value	OR (95% CI)
BASDAI	0.80	0.37	0	9.50	<0.00001	−2.40 (−2.89, −1.9)
ST	7.73	0.02	74	2.56	0.01	0.86 (0.20 1.52)
CRP	7.29	0.03	73	4.22	<0.0001	−8.01 (−11.73, −4.29)

BASDAI, Bath ankylosing spondylitis disease activity index; ST, Schober’s test; CRP, C-reactive protein; OR, odds ratio; CI, confidence interval.

## References

[1] Braun J, Sieper J (2007). Ankylosing spondylitis. Lancet.

[2] Feldtkeller E, Khan M, van der Heijde D (2003). Age at disease onset and diagnosis delay in HLA-B27 negative vs. positive patients with ankylosing spondylitis. Rheumatol Int.

[3] Sieper J, Braun J (2002). Anti-TNF agents for the treatment of spondyloarthropathies. Expert Opin Emerg Drugs.

[4] Gratacos J, Collado A, Filella X (1994). Serum cytokines (IL-6, TNF-α, IL-1β and IFN-γ) in ankylosing spondylitis: a close correlation between serum IL-6 and disease activity and severity. Rheumatology.

[5] Braun J, Bollow M, Neure L (1995). Use of immunohistologic and in situ hybridization techniques in the examination of sacroiliac joint biopsy specimens from patients with ankylosing spondylitis. Arthritis Rheum.

[6] Culy CR, Keating GM (2002). Etanercept: an updated review of its use in rheumatoid arthritis, psoriatic arthritis and juvenile rheumatoid arthritis. Drugs.

[7] Mease PJ, Goffe BS, Metz J (2000). Etanercept in the treatment of psoriatic arthritis and psoriasis: a randomised trial. Lancet.

[8] Lovell DJ, Giannini EH, Reiff A (2000). Etanercept in children with polyarticular juvenile rheumatoid arthritis. N Engl J Med.

[9] Braun J, Van den Berg R, Baraliakos X (2011). 2010 update of the ASAS/EULAR recommendations for the management of ankylosing spondylitis. Ann Rheum Dis.

[10] Chen J, Liu C (2005). Sulfasalazine for ankylosing spondylitis. Cochrane Database Syst Rev.

[11] Ferraz MB, Tugwell P, Goldsmith CH (1990). Meta-analysis of sulfasalazine in ankylosing spondylitis. J Rheumatol.

[12] Li SH, Ma B, Tan JY (2009). Efficacy and safety of etanercept for patients with ankylosing spondylitis: a systematic review. Chinese Journal of Evidence-Based Medicine.

[13] Moher D, Liberati A, Tetzlaff J, Altman DG, PRISMA Group (2009). Preferred reporting items for systematic reviews and meta-analyses: the PRISMA statement. Ann Intern Med.

[14] van der Linden S, Valkenburg HA, Cats A (1984). Evaluation of diagnostic criteria for ankylosing spondylitis. A proposal for modification of the New York criteria. Arthritis Rheum.

[15] Jadad AR, Moore RA, Carroll D (1996). Assessing the quality of reports of randomized clinical trials: is blinding necessary?. Control Clin Trials.

[16] Anderson JJ, Baron G, van der Heijde D (2001). Ankylosing spondylitis assessment group preliminary definition of short-term improvement in ankylosing spondylitis. Arthritis Rheum.

[17] Garrett S, Jenkinson T, Kennedy LG (1994). A new approach to defining disease status in ankylosing spondylitis: the Bath Ankylosing Spondylitis Disease Activity Index. J Rheumatol.

[18] Calin A, Garrett S, Whitelock H (1994). A new approach to defining functional ability in ankylosing spondylitis: the development of the Bath Ankylosing Spondylitis Functional Index. J Rheumatol.

[19] Dougados M, Braun J, Szanto S (2011). Efficacy of etanercept on rheumatic signs and pulmonary function tests in advanced ankylosing spondylitis: results of a randomised double-blind placebo-controlled study (SPINE). Ann Rheum Dis.

[20] Lin Q, Lin Z, Gu J (2010). Abnormal high-expression of CD154 on T lymphocytes of ankylosing spondylitis patients is down-regulated by etanercept treatment. Rheumatol Int.

[21] Gorman JD, Sack KE, Davis JC (2002). Treatment of ankylosing spondylitis by inhibition of tumor necrosis factor α. N Engl J Med.

[22] Davis JC, Van Der Heijde D, Braun J, Enbrel Ankylosing Spondylitis Study Group (2003). Recombinant human tumor necrosis factor receptor (etanercept) for treating ankylosing spondylitis: a randomized, controlled trial. Arthritis Rheum.

[23] Braun J, van der Horst-Bruinsma IE, Huang F (2011). Clinical efficacy and safety of etanercept versus sulfasalazine in patients with ankylosing spondylitis: A randomized, double-blind trial. Arthritis Rheum.

[24] Calin A, Dijkmans BAC, Emery P (2004). Outcomes of a multicentre randomised clinical trial of etanercept to treat ankylosing spondylitis. Ann Rheum Dis.

[25] van der Heijde D, Da Silva JC, Dougados M, Etanercept Study 314 Investigators (2006). Etanercept 50 mg once weekly is as effective as 25 mg twice weekly in patients with ankylosing spondylitis. Ann Rheum Dis.

[26] Brandt J, Khariouzov A, Listing J (2003). Six-month results of a double-blind, placebo-controlled trial of etanercept treatment in patients with active ankylosing spondylitis. Arthritis Rheum.

[27] Huang F, Zhang J, Zheng Y (2011). A multicenter, double-blind, randomized, placebo-controlled clinical trial of etanercept in the treatment of Chinese patients with active ankylosing spondylitis. Zhonghua Nei Ke Za Zhi.

[28] Huang F, Zhang J, Huang JL (2010). A multicenter, double-blind, placebo-controlled, randomized clinical study of etanercept in the treatment of ankylosing spondylitis. Zhonghua Nei Ke Za Zhi.

[29] Deng XH, Hang F, Zhang YM (2009). Treatment of ankylosing spondylitis with recombinant human tumor necrosis factor-Fc fusion protein (etanercept): a multicenter, randomized, double-blind, placebo-controlled trial. Jun Yi Jin Xiu Xue Yuan Xue Bao.

[30] Zhang J, Zhang YM, Zhang JL (2009). Efficacy of etanercept in patients with ankylosing spondylitis: A double-blind, randomized, placebo controlled trial. Zhongguo Xin Yao Za Zhi.

[31] Zhao WM, Chen ZW, Wang MJ (2009). Clinical observation of etanercept in treatment of ankylosing spondylitis. Suzhou Da Xue Xue Bao.

[32] Zhao FT, Zhao H, Wang YL (2009). Efficacy of etanercept on ankylosing spondylitis. Shanghai Jiaotong Daxue Xuebao Yixueban.

[33] Sterne JA, Gavaghan D, Egger M (2000). Publication and related bias in meta-analysis: power of statistical tests and prevalence in the literature. J Clin Epidemiol.

[34] Davis JC (2002). The role of etanercept in ankylosing spondylitis. Clin Exp Rheumatol.

[35] Chen MZ, Zhao RGT, Wang HY (2010). Clinical control study of recombinant human tumor necrosis factor-Fc fusion protein and traditional immunity depressant in treatment of ankylosing spondylitis (AS). Xinjiang Yi Ke Da Xue Xue Bao.

[36] Machado MA, Barbosa MM, Almeida AM (2013). Treatment of ankylosing spondylitis with TNF blockers: a meta-analysis. Rheumatol Int.

[37] Li ZH, Zhang Y, Wang J, Shi ZJ (2013). Etanercept in the treatment of ankylosing spondylitis: a meta-analysis of randomized, double-blind, placebo-controlled clinical trials, and the comparison of the Caucasian and Chinese population. Eur J Orthop Surg Traumatol.

